# Supplementary material for: Socio-ecological risk factors associated with human flea infestations of rural household in plague-endemic areas of Madagascar
Source: PLoS Negl Trop Dis. 2024 Mar 7;18(3):e0012036. doi: 10.1371/journal.pntd.0012036 (PMC10950221; doi:10.1371/journal.pntd.0012036)
Supplement: S1 File — (PDF) [file pntd.0012036.s001.pdf]

## Household survey form

District:
Commune:
Fokontany:

Village name:
Total household number in the village:
Village GPS coordinate:
GPS Long.:
GPS Lat.:
Elevation:

Enumerator name:
Date of the interview:
Household code:

### Declaration of Informed Consent

***Instruction to Enumerator***: *please read the declaration of informed consent document and only proceed when all the answers with the consent questions are "yes".*

Oral consent: The Head of the Household is willing to participate in the survey.

Yes ☐ No ☐

---

## Section 1. Observation

### Observation from the outside of the main house

***Instructions to Enumerator:*** *please ask the permission to look around the main house.*

1.1. Closest distance (radius) from another household:
0-1 m ☐ 2-3 m ☐ 4-5 m ☐ > 5m ☐

1.2. Presence of farm animal housing around the main house:
Yes ☐ (Continue to 1.3 and 1.4) No ☐ (continue to 1.5)

1.3. Animal housing type:
Zebu enclosure (no roof) ☐ Stable ☐ Coop ☐ Pigpen (hog house) ☐ Other ☐: _______________

1.4. Closest distance (radius) from animal housing to the main house:
0-1 m ☐ 2 -3 m ☐ 4-5 m ☐ >5 m ☐

1.5. Presence of rodent holes and dropping:
Yes ☐ No ☐

1.6. The house is constructed on elevated platform:
Yes ☐ No ☐

1.7. Number of floors (story):
***Instructions to Enumerator:*** *"0" story means that there is no upper level in the house but only ground floor*
0 ☐ 1 ☐ 2 ☐ 3 ☐ 4 ☐ >4 ☐

1.8. Roof:
No roof ☐ Thatch/Palm leaves ☐ Metal sheet ☐ Tiles ☐ Other: ____________________

### Observation of the room where the candle is placed

***Instructions to Enumerator***: *can be done before or after the interview, after explaining how to use the candle trap. Please enter the room and note what you see from the inside.*

1.9. In which story # did you put the candle trap? *(Story# 0 is ground floor and so on)*
#0☐ 1 ☐ 2 ☐ 3 ☐ 4 ☐ >4 ☐

1.10. What type of wall do you observe in the room where the candle is placed?

No wall ☐ Bamboo/Cane/ Palm/Tree Trunk ☐ Wooden boards ☐
Mud ☐ Bamboo and mud ☐ Stone and mud ☐ Brick ☐ Cardboard ☐
Plywood ☐ Cement ☐ Other: ________________

1.11. What type of ceiling do you observe in the room where the candle is placed?
No ceiling (you can see the roof) ☐ Mat ☐ Bamboo ☐ Mud ☐ Mud and bamboo ☐ Cardboard ☐ Wooden boards ☐ Cement ☐
Other:__________________

1.12. What type of floor do you observe in the room where the candle is placed?
Dirt/ sand/ Mud ☐ Manure/Dung ☐ Mat ☐ Tile ☐ Carpet ☐
Cement ☐ Wooden boards ☐ Other: ___________________

1.13. How many Windows do you observe in the room where the candle is placed?
**number**: _ _

1.14. Is there a stove or cooking place in the room where the candle is placed?
Yes ☐ No ☐

1.15. Do you observe any stored crops and/ or food in the room where the candle is placed?
None ☐ Unmilled rice ☐ Milled rice ☐
Beans ☐ Corn ☐ Potatoes ☐ Other :_________________

1.16. Is there any rodent hole or/ and rodent droppings in the room where the candle is placed?
Rodent hole number: _ _ Rodent dropping: Yes ☐ No ☐

---

## Section 2. Information from each member of the household

***Instructions to Enumerator:*** *important note: the numbers (#) assigned to each individual in this section should be retained throughout the survey. Please make a note of the number assigned to each individual so that you can reference them in later questions.*
*Please record the responses given by the Head of Household (HH) for each question. If the response provided does not match an option provided, check 'Other' and clearly specify the specific response given. UK: unknown.*

### 2.1. Household demographics

***Instructions to Enumerator****: individuals belonging to the category "permanent" are considering the house as their first residence, in opposition to individuals who sleep in this residence for a limited time of the year such as for family events, work or other reasons. Individuals having family ties with the Head of the Household (HH) but sleeping in other homes are not considered part of the household for this study purpose.*

2.1.1. We would like to make an inventory of the household members. Could you please tell me the size of the household (the number of person)?
Number: _ _ Continue to 2.1.2.

2.1.2. What is the relation of each household member with the HH?
***Instructions to Enumerator****: circle the number corresponding to the person to be interviewed*

| | #1 | #2 | #3 | #4 | #5 | #6 | #7 | #8 |
|---|---|---|---|---|---|---|---|---|
| HH | ☐ | ☐ | ☐ | ☐ | ☐ | ☐ | ☐ | ☐ |
| Spouse/Partner of HH | ☐ | ☐ | ☐ | ☐ | ☐ | ☐ | ☐ | ☐ |
| Child of HH | ☐ | ☐ | ☐ | ☐ | ☐ | ☐ | ☐ | ☐ |
| Grandchild of HH | ☐ | ☐ | ☐ | ☐ | ☐ | ☐ | ☐ | ☐ |
| Parent or In-law of HH | ☐ | ☐ | ☐ | ☐ | ☐ | ☐ | ☐ | ☐ |
| Sibling of HH | ☐ | ☐ | ☐ | ☐ | ☐ | ☐ | ☐ | ☐ |
| Immediate cousin of HH | ☐ | ☐ | ☐ | ☐ | ☐ | ☐ | ☐ | ☐ |

| | | | | | | | | |
|---|---|---|---|---|---|---|---|---|
| Aunt/uncle of the HH | ☐ | ☐ | ☐ | ☐ | ☐ | ☐ | ☐ | ☐ |
| Niece/nephew of the HH | ☐ | ☐ | ☐ | ☐ | ☐ | ☐ | ☐ | ☐ |
| No biological /familial relation | ☐ | ☐ | ☐ | ☐ | ☐ | ☐ | ☐ | ☐ |
| Other: ______________________ | ☐ | ☐ | ☐ | ☐ | ☐ | ☐ | ☐ | ☐ |

Continue to 2.1.3.

2.1.3. How old is every member of the household?

| Indiv. # | #1 | #2 | #3 | #4 | #5 | #6 | #7 | #8 |
|---|---|---|---|---|---|---|---|---|
| Age: | __ | __ | __ | __ | __ | __ | __ | __ |

Continue to 2.1.4.

***Instructions to Enumerator:*** *use > or < of the closest age if not known. For infants less than 1 year old, mark the # of month with the letter M.*

2.1.4. What is the sex of each member of the household?

| Indiv. # | #1 | #2 | #3 | #4 | #5 | #6 | #7 | #8 |
|---|---|---|---|---|---|---|---|---|
| sex: | M ☐ | M ☐ | M ☐ | M ☐ | M ☐ | M ☐ | M ☐ | M ☐ |
| | F ☐ | F ☐ | F ☐ | F ☐ | F ☐ | F ☐ | F ☐ | F ☐ |

Continue to 2.1.5.

2.1.5. Which household member is living permanently in this household?

#1 ☐ #2 ☐ #3 ☐ #4 ☐ #5 ☐ #6 ☐ #7 ☐ #8 ☐ Continue to 2.2.

## 2.2. Sleeping arrangement

2.2.1. How many rooms are used as bedroom?

Number: _ _ Continue to 2.2.2.

2.2.2. Which household members are currently sleeping in each room?

***Instructions to Enumerator****: ask which individuals are sleeping in the same room, and then ask the floor number and room number. Room number assignment is arbitrary but allow us to know the number of individual sleeping in each room.*

| Individual number | #1 | #2 | #3 | #4 | #5 | #6 | #7 | #8 |
|---|---|---|---|---|---|---|---|---|
| Floor number | __ | __ | __ | __ | __ | __ | __ | __ |
| Room number | __ | __ | __ | __ | __ | __ | __ | __ |

Continue to 2.2.3.

2.2.3. Are there any household members sleeping on the floor (not on elevated bed)?

Yes ☐ Continue to 2.2.4. No ☐ Continue to 2.2.5.

2.2.4. Which household members are currently sleeping on elevated bed or on the floor?

| | #1 | #2 | #3 | #4 | #5 | #6 | #7 | #8 |
|---|---|---|---|---|---|---|---|---|
| On the floor | ☐ | ☐ | ☐ | ☐ | ☐ | ☐ | ☐ | ☐ |
| Elevated bed | ☐ | ☐ | ☐ | ☐ | ☐ | ☐ | ☐ | ☐ |

Continue to 2. 2.5.

2.2.5. What type of mattress each household member is sleeping on?

| | #1 | #2 | #3 | #4 | #5 | #6 | #7 | #8 |
|---|---|---|---|---|---|---|---|---|
| No mattress or mat: | ☐ | ☐ | ☐ | ☐ | ☐ | ☐ | ☐ | ☐ |
| Mat | ☐ | ☐ | ☐ | ☐ | ☐ | ☐ | ☐ | ☐ |
| Dried vegetal fibers stuffed in a sac | ☐ | ☐ | ☐ | ☐ | ☐ | ☐ | ☐ | ☐ |
| Foam | ☐ | ☐ | ☐ | ☐ | ☐ | ☐ | ☐ | ☐ |
| Other: ___________________________ | ☐ | ☐ | ☐ | ☐ | ☐ | ☐ | ☐ | ☐ |

Continue 2.2.6.

2.2.6. Is insecticide treated bed net used in this household?

Yes ☐ Continue to 2.2.7. No ☐ Continue to 2.2.9.

2.2.7. Which household member is sleeping under insecticide treated bed net?

#1 ☐ #2 ☐ #3☐ #4 ☐ #5☐ #6 ☐ #7 ☐ #8 ☐

2.2.8. When did you receive the insecticide treated bed net?

UK ☐ Open: ________________________________________________-__Continue to 2.2.9

2.2.9. Are there any household members sleeping in a room with cooking stove?

Yes ☐ Continue to 2.2.10. No ☐ Continue to 2.2.11.

2.2.10. Which household member is sleeping in a room with a cooking stove?

#1 ☐ #2 ☐ #3☐ #4 ☐ #5☐ #6 ☐ #7 ☐ #8 ☐ Continue to 2.2.11.

2.2.11. Are there any household members sleeping in a room with stored food or crop?

Yes ☐ Continue to 2.2.12. No ☐ Continue to 2.2.13.

2.2.12. Which household member is sleeping in a room with stored food or crop?

#1 ☐ #2 ☐ #3☐ #4 ☐ #5☐ #6 ☐ #7 ☐ #8 ☐ Continue to 2.2.13.

2.2.13. Is the candle trap placed in a bedroom?

Yes ☐ Continue to 2.2.14. No ☐ Continue to 2.3.

2.2. 14. Which household member is sleeping in the room where the candle trap is?

#1 ☐ #2 ☐ #3☐ #4 ☐ #5☐ #6 ☐ #7 ☐ #8 ☐ Continue to 2.3.

## 2.3. Household socioeconomics

2.3.1. Which individuals are working?

#1 ☐ #2 ☐ #3☐ #4 ☐ #5☐ #6 ☐ #7 ☐ #8 ☐ Continue to 2.3.2.

2.3.2. How are working individuals paid? (What form of compensation do they earn?)

| | #1 | #2 | #3 | #4 | #5 | #6 | #7 | #8 |
|---|---|---|---|---|---|---|---|---|
| Money | ☐ | ☐ | ☐ | ☐ | ☐ | ☐ | ☐ | ☐ |
| Kind | ☐ | ☐ | ☐ | ☐ | ☐ | ☐ | ☐ | ☐ |
| Money and kind | ☐ | ☐ | ☐ | ☐ | ☐ | ☐ | ☐ | ☐ |
| Not paid | ☐ | ☐ | ☐ | ☐ | ☐ | ☐ | ☐ | ☐ |

2.3.3 What is the principal activity of everyone in this household?

| Activity | #1 | #2 | #3 | #4 | #5 | #6 | #7 | #8 |
|---|---|---|---|---|---|---|---|---|
| Student | ☐ | ☐ | ☐ | ☐ | ☐ | ☐ | ☐ | ☐ |
| Caretaker/homemaker | ☐ | ☐ | ☐ | ☐ | ☐ | ☐ | ☐ | ☐ |
| Agri./pastoralist | ☐ | ☐ | ☐ | ☐ | ☐ | ☐ | ☐ | ☐ |
| Civil servant | ☐ | ☐ | ☐ | ☐ | ☐ | ☐ | ☐ | ☐ |
| Business/trade/industry | ☐ | ☐ | ☐ | ☐ | ☐ | ☐ | ☐ | ☐ |
| Art and crafts | ☐ | ☐ | ☐ | ☐ | ☐ | ☐ | ☐ | ☐ |
| Extraction/mining | ☐ | ☐ | ☐ | ☐ | ☐ | ☐ | ☐ | ☐ |
| Field assistant | ☐ | ☐ | ☐ | ☐ | ☐ | ☐ | ☐ | ☐ |
| Healthcare | ☐ | ☐ | ☐ | ☐ | ☐ | ☐ | ☐ | ☐ |
| Education | ☐ | ☐ | ☐ | ☐ | ☐ | ☐ | ☐ | ☐ |
| Other: _____________ | ☐ | ☐ | ☐ | ☐ | ☐ | ☐ | ☐ | ☐ |

Continue 2.3.4.

2.3.4. What is the highest education grade completed by each member of the household?

| Education level: | #1 | #2 | #3 | #4 | #5 | #6 | #7 | #8 |
|---|---|---|---|---|---|---|---|---|
| Never went to school | ☐ | ☐ | ☐ | ☐ | ☐ | ☐ | ☐ | ☐ |
| Did not finish primary school | ☐ | ☐ | ☐ | ☐ | ☐ | ☐ | ☐ | ☐ |

| | | | | | | | | |
|---|---|---|---|---|---|---|---|---|
| Get the CEPE | ☐ | ☐ | ☐ | ☐ | ☐ | ☐ | ☐ | ☐ |
| Did not finish middle school | ☐ | ☐ | ☐ | ☐ | ☐ | ☐ | ☐ | ☐ |
| Get the BEPC | ☐ | ☐ | ☐ | ☐ | ☐ | ☐ | ☐ | ☐ |
| Did not finish high school | ☐ | ☐ | ☐ | ☐ | ☐ | ☐ | ☐ | ☐ |
| Get the Bacc | ☐ | ☐ | ☐ | ☐ | ☐ | ☐ | ☐ | ☐ |
| Went to College | ☐ | ☐ | ☐ | ☐ | ☐ | ☐ | ☐ | ☐ |
| Get College diploma | ☐ | ☐ | ☐ | ☐ | ☐ | ☐ | ☐ | ☐ |

Continue to section 3.

---

## Section 3. Domestic animals

3.1. Do you have dogs living or roaming on your property?

Yes ☐ Continue to 3.2. No ☐ Continue to 3.4. UK ☐ Continue to 3.4.

3.2. How many dogs do you have or observe on your property?

Number: _ _ Continue to 3.3.

3.3. Within the last two months, how frequent did you see any dogs inside the main house, during the day or night?

Never☐ Sometimes ☐ Always ☐ UK ☐ Continue to 3.4.

3.4. Do you have cats living or roaming on your property?

Yes ☐ Continue to 3.5. No ☐ Continue to 3.7. UK ☐ Continue to 3.4.

3.5. How many cats do you have or observe on your property?

Number: _ _ Continue to 3.7.

3.6. Within the last two months, how frequent did you see any cats inside the main house, during the day or night?

Never☐ Sometimes ☐ Always ☐ UK ☐ Continue to 3.7.

3.7.1 Do you own any domestic animals?

Yes ☐ Continue to 3.7.2 No ☐ Continue to section 4

3.7.2 Which animals do you own, how many do you own and where do you keep them? Additionally, for animals kept inside, does anyone sleep in the room(s) where these animals are kept?

Continue to section 4

| Animals | Where are they housed? (Check all that apply) | | | Number of animals | Who sleeps in the room(s) where they are kept? (List identity #'s) | Optional notes |
|---|---|---|---|---|---|---|
| | Outside only | Inside Day | Inside Night | | | |
| Chickens | ☐ | ☐ | ☐ | _ _ | | |
| Pigs | ☐ | ☐ | ☐ | _ _ | | |
| Cows | ☐ | ☐ | ☐ | _ _ | | |
| Ducks | ☐ | ☐ | ☐ | _ _ | | |
| Goose | ☐ | ☐ | ☐ | _ _ | | |
| Rabbits | ☐ | ☐ | ☐ | _ _ | | |
| Guinea Pigs | ☐ | ☐ | ☐ | _ _ | | |
| Goat | ☐ | ☐ | ☐ | _ _ | | |
| Sheep | ☐ | ☐ | ☐ | _ _ | | |
| Fowl | ☐ | ☐ | ☐ | _ _ | | |

| Other | ☐ | ☐ | ☐ | _ _ | | |
|---|---|---|---|---|---|---|

Continue to section 4

## Section 4. Household habits

***Instructions to Enumerator**: the following questions are focusing on household habits in the room where the candle trap is placed. Check all that apply and continue as directed*

4.1. What is done in your household to maintain the floor clean?

| Actions → Floor type: | Sweep → 4.2. | Brush → 4.3. | Mop → 4.4. | Wax →4.5. | Other → 4.6. |
|---|---|---|---|---|---|
| Mud/sand/dust | ☐ | ☐ | ☐ | ☐ | ☐ |
| Manure/dung | ☐ | ☐ | ☐ | ☐ | ☐ |
| Mat | ☐ | ☐ | ☐ | ☐ | ☐ |
| Carpet | ☐ | ☐ | ☐ | ☐ | ☐ |
| Board | ☐ | ☐ | ☐ | ☐ | ☐ |
| Cement | ☐ | ☐ | ☐ | ☐ | ☐ |
| Tile | ☐ | ☐ | ☐ | ☐ | ☐ |
| Other | ☐ | ☐ | ☐ | ☐ | ☐ |

4.2. How often the floor is swept?
Daily ☐ Weekly ☐ Occasionally ☐ Rarely ☐ Continue to 4.8.
4.3. How often the floor is brushed?
Daily ☐ Weekly ☐ Occasionally ☐ Rarely ☐ Continue to 4.8.
4.4. How often the floor is mopped?
Daily ☐ Weekly ☐ Occasionally ☐ Rarely ☐ Continue to 4.8.
4.5. How often the floor is waxed?
Daily ☐ Weekly ☐ Occasionally ☐ Rarely ☐ Continue to 4.8.
4.6. Other: ________________________ Continue to 4.7.
4.7. How often?
Daily ☐ Weekly ☐ Occasionally ☐ Rarely ☐ Continue to 4.8.
4.8. Is the furniture moved when cleaning the floor?
Yes ☐ No ☐ UK ☐ Continue to 4.9.
4.9. In the last two months, were the windows left open during the day?
Yes ☐ No ☐ UK ☐ Continue to 4.10.
4.10. In the last two months, was the main door usually left open during the day?
Yes ☐ No ☐ UK ☐ Continue to 4.11.
4.11. Within the last two months, was the house deserted at any moment*?
Yes ☐ continue to 4.12. No ☐ Continue to section 5. UK ☐ Continue to section 5.4.12. How long did it last?
Open: ___________________________________________________________________________________

4.13 What type of fuel is mainly used for cooking?

Wood ☐ Coal/charcoal ☐ crop residue¨☐ Garbage/plastic ☐¨ Other: ____________________________ Continue to

4.14 When the stove generates smoke, what do you do about the smoke?

Nothing ☐ Open windows/doors ☐ Use chimney, vent, or fan ☐¨

Other: ___________________ Continue to section 5

**Section 5. Pest control and insecticide use in general.**

***Instructions to Enumerator****: we want to know if certain behaviors toward pest nuisance perception and the use of pest control techniques could have an impact flea abundance*.

5.1. Do you experience pest nuisance in your home?

Yes ☐ (continue to 5.2.) No ☐ (Continue to 5.3.)

5.2. What kind of pest do you observe in your home?

Fleas ☐ Mosquitoes☐ Cockroaches ☐ Bedbugs ☐ Lice ☐

Other: _______________________

5.3. Have you ever taken any measure to address pest nuisance in your home?

Yes ☐ continue to 5.4. No ☐ Continue to section 6.

5.4. What measure do you take to address each pest nuisance?

***Instructions to Enumerator****: "pesticides" are chemicals they buy from store or market. The use of soap and plant are among the "other measures" category*

Pesticides ☐ Continue to 5.5. Other measures ☐ Continue to 6.6

5.5. What is the name of the pesticide that you used?

(1): _____________________ (2): ________________________ Continue to 5.7.

5.6. What are the other measures that you used to manage pest nuisance at home?

(A): ___________________________________________________ Continue to 5.7.

(B): ___________________________________________________

5.7. What pesticide form do you use?

| | Powder | Liquid | Spray | Coils | Other |
|---|---|---|---|---|---|
| (1) | ☐ | ☐ | ☐ | ☐ | ☐ |
| (2) | ☐ | ☐ | ☐ | ☐ | ☐ |
| (A) | ☐ | ☐ | ☐ | ☐ | ☐ |
| (B) | ☐ | ☐ | ☐ | ☐ | ☐ |

Other: ______________________________________________________________ Continue to 5.8.

5.8. Against what kind of pest do you take those measures?

| | Fleas | Mosquitoes | Cockroaches | Bedbugs | Lice | Others: _____________ |
|---|---|---|---|---|---|---|
| (1) | ☐ | ☐ | ☐ | ☐ | ☐ | ☐ |
| (2) | ☐ | ☐ | ☐ | ☐ | ☐ | ☐ |
| (A) | ☐ | ☐ | ☐ | ☐ | ☐ | ☐ |
| (B) | ☐ | ☐ | ☐ | ☐ | ☐ | ☐ |

Continue to 5.9.

5.9. How often do you use those measures?

| | Daily | Weekly | Often | Rarely | Other: _________________ |
|---|---|---|---|---|---|
| (1) | ☐ | ☐ | ☐ | ☐ | ☐ |
| (2) | ☐ | ☐ | ☐ | ☐ | ☐ |
| (A) | ☐ | ☐ | ☐ | ☐ | ☐ |
| (B) | ☐ | ☐ | ☐ | ☐ | ☐ |

Continue to 5.9.

5.10. In the past two months, when did you use those measures for the last time?

| | (1) | (2) | (A) | (B) |
|---|---|---|---|---|
| Did not use | ☐ | ☐ | ☐ | ☐ |
| Less than a week ago | ☐ | ☐ | ☐ | ☐ |
| More than one week but less than one month ago | ☐ | ☐ | ☐ | ☐ |
| More than one month ago | ☐ | ☐ | ☐ | ☐ |

| UK | | ☐ | ☐ | ☐ | ☐ | Continue to 5.11. |
|---|---|---|---|---|---|---|

5.11. Which one of the rooms do you usually treat with those measures?

| | All rooms | Bedrooms | Kitchen | Rooms with animals | Other |
|---|---|---|---|---|---|
| (1) | ☐ | ☐ | ☐ | ☐ | ☐ |
| (2) | ☐ | ☐ | ☐ | ☐ | ☐ |
| (A) | ☐ | ☐ | ☐ | ☐ | ☐ |
| (B) | ☐ | ☐ | ☐ | ☐ | ☐ |

Other: ________________________________________________________ Continue to 5.12.

5.12. Where in the room do you put the product?

| | On the wall | On the floor | On the furniture | Under the furniture | Other |
|---|---|---|---|---|---|
| (1) | ☐ | ☐ | ☐ | ☐ | ☐ |
| (2) | ☐ | ☐ | ☐ | ☐ | ☐ |
| (A) | ☐ | ☐ | ☐ | ☐ | ☐ |
| (B) | ☐ | ☐ | ☐ | ☐ | ☐ |

Other: _______________________________________________________ Continue to 5.13.

5.13. How would you qualify the efficacy of the measure you take to eliminate pest nuisance?

| | Not efficient at all | Slightly efficient | Very efficient | UK |
|---|---|---|---|---|
| (1) | ☐ | ☐ | ☐ | ☐ |
| (2) | ☐ | ☐ | ☐ | ☐ |
| (A) | ☐ | ☐ | ☐ | ☐ |
| (B) | ☐ | ☐ | ☐ | ☐ |

Continue to 5.14.

5.14. How long does the efficacy of the measure you take against pest nuisance last?

| | Days | Week | Months | Years | Other: ___________ |
|---|---|---|---|---|---|
| (1) | ☐ | ☐ | ☐ | ☐ | ☐ |
| (2) | ☐ | ☐ | ☐ | ☐ | ☐ |
| (A) | ☐ | ☐ | ☐ | ☐ | ☐ |
| (B) | ☐ | ☐ | ☐ | ☐ | ☐ |

Continue to 5.14.

---

## Section 6. Flea nuisance and usual practices for flea control

6.1. Have you or someone in your household ever experience flea bite inside your home?

Yes ☐ continue to 6.2. No ☐ Continue to 6 section 7.

6.2. How often are you or your household members experience flea bites?

Never ☐ Rarely ☐ Always ☐ Constantly ☐ UK ☐ Continue to 6.3.

6.3. During what time of day do you notice the most flea bites/nuisance?

Morning ☐ Afternoon ☐ Evening ☐ At night ☐ UK ☐ Continue to 6.4

6.4. Where in the house do you usually experience most of flea bite?

Open: ____________________ UK ☐ Continue to 6.5.

6.5. During what months of year do you notice the most flea bites/nuisance?

Open: ________________________________________ Continue to 6.6.

6.6. Why do you think you see fleas the most during that time?

Open: ________________________________________

Continue to section 7.

---

**Section 7. Rodent presence and perception**

7.1. In the past two months, have you ever observe/ hear rodents in your home?
Yes ☐ continue to 7.2. No ☐ Continue to section 7.4.
7.2. In the past two months, how often did you observe/hear rodents in your home?
Rarely ☐ Often ☐ Usually ☐ Always ☐ Continue to 7.3.
7.3. In the past two months, which one of these small mammals have you seen in your home (select all that apply)?
Mouse ☐ Rat ☐ Shrew ☐ UK ☐ Continue to 7.3.
7.4. In the past two months, have you ever observe/ hear rodents around your home (outdoor)?
Yes ☐ Continue to 7.5. No ☐ Continue to 7.5.
7.5. In the past two months, have you ever seen dead rodent inside your home or around your house?
Yes, indoor ☐ No, indoor ☐
Yes, outdoor ☐ No, outdoor ☐ Continue to 7.6.
7.6. In the past two months, have you ever observe/ hear rodents within the village?
Yes ☐ Continue to 7.7. No ☐ Continue to section 8.
7.7. In the past two months, have you ever observe/ hear rodents within the village?
Open: ____________________________________________________________Continue to section 8
_____________________________________________________________________________________

**<u>Section 8. Household Socioeconomics</u>**

***Instructions to Enumerator:*** *the following questions are designed to assess the household's wealth and well-being, which will help us determine if flea infestation is directly related to poverty.*

8.1. How many bags of rice did your household produce in the last year?
Number: __ UK ☐ Did not produce ☐ Continue to 8.2.
8.2. How much did your household spend (in Ariary) on healthcare in the last year?
Ar _________ UK ☐ Continue to 8.3.
8.3. Do members of this household have a farmland?
Yes ☐ continue to 8.4 No ☐ End of the questionnaire
End of the questionnaire
