# Supplementary material for: Socio-ecological risk factors associated with human flea infestations of rural household in plague-endemic areas of Madagascar
Source: PLoS Negl Trop Dis. 2024 Mar 7;18(3):e0012036. doi: 10.1371/journal.pntd.0012036 (PMC10950221; doi:10.1371/journal.pntd.0012036)

Additional file 2

Traditional three-story house in the central highland of Madagascar with various roof type. Photo credit: Adelaide Miarinjara

(1) Thatched roof

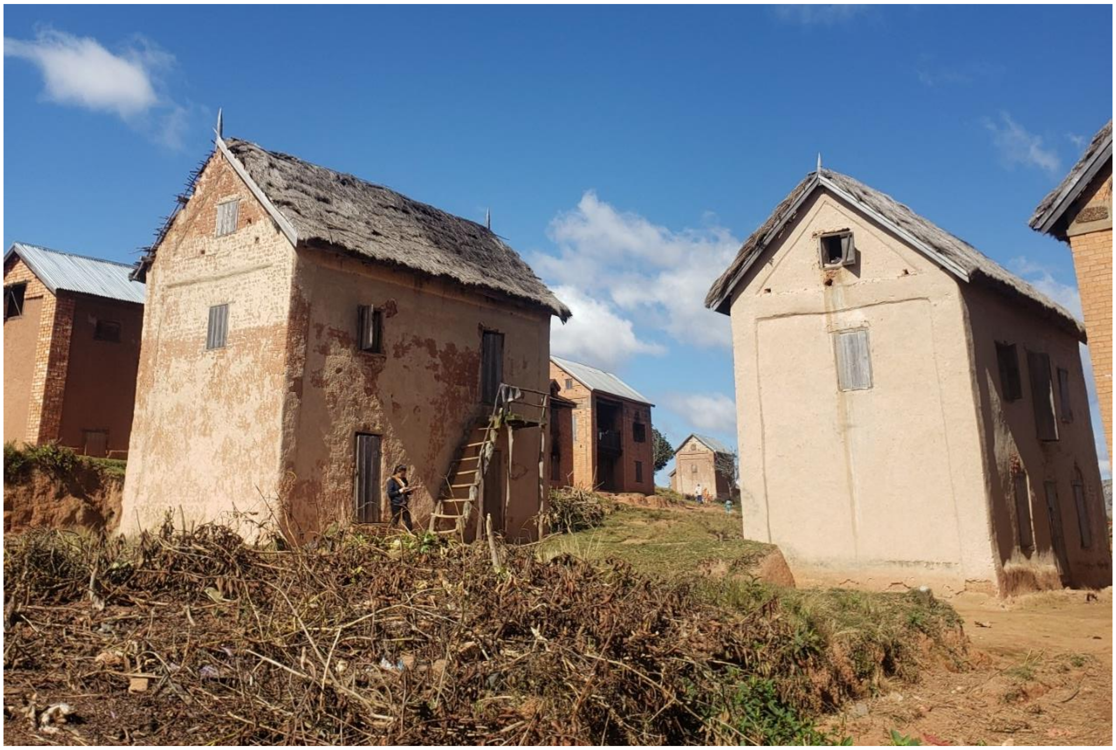

(2) Clay tiles

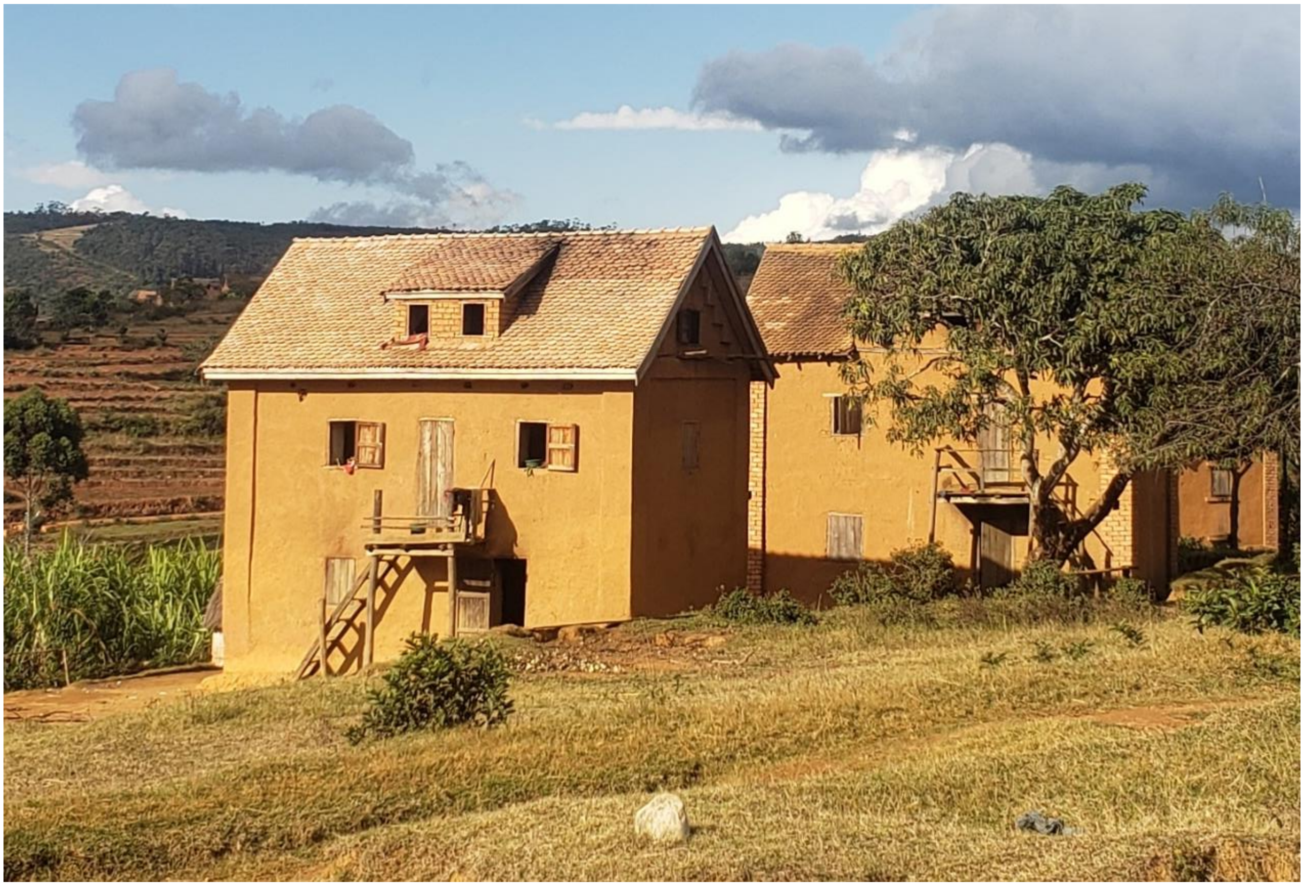

(3) Metal sheet

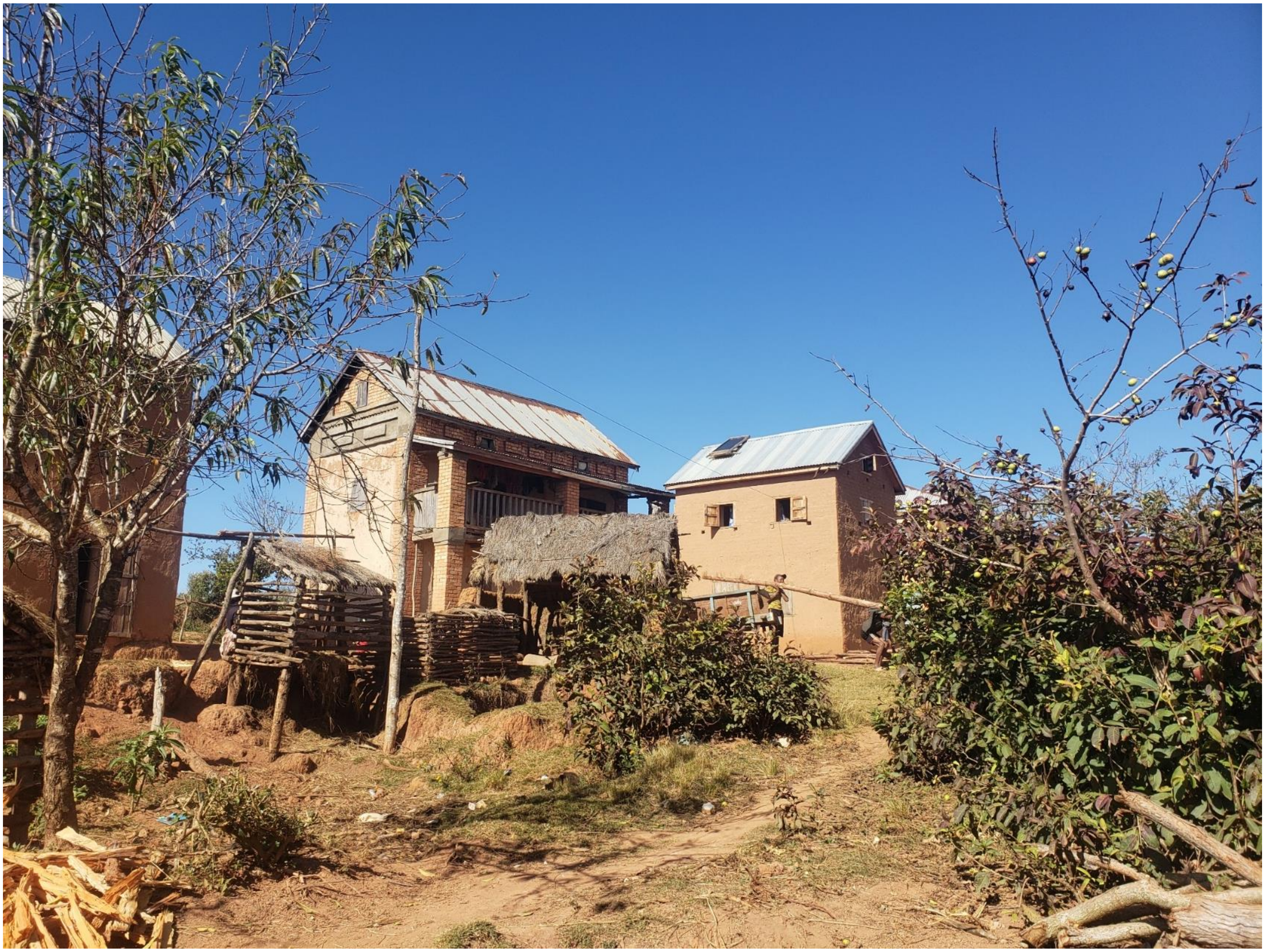

Supplement: S2 File — (PDF) [file pntd.0012036.s002.pdf]
