# Supplementary material for: Socio-ecological risk factors associated with human flea infestations of rural household in plague-endemic areas of Madagascar
Source: PLoS Negl Trop Dis. 2024 Mar 7;18(3):e0012036. doi: 10.1371/journal.pntd.0012036 (PMC10950221; doi:10.1371/journal.pntd.0012036)

Diagram of three-story traditional house in the central highland of Madagascar, with common use of each level.

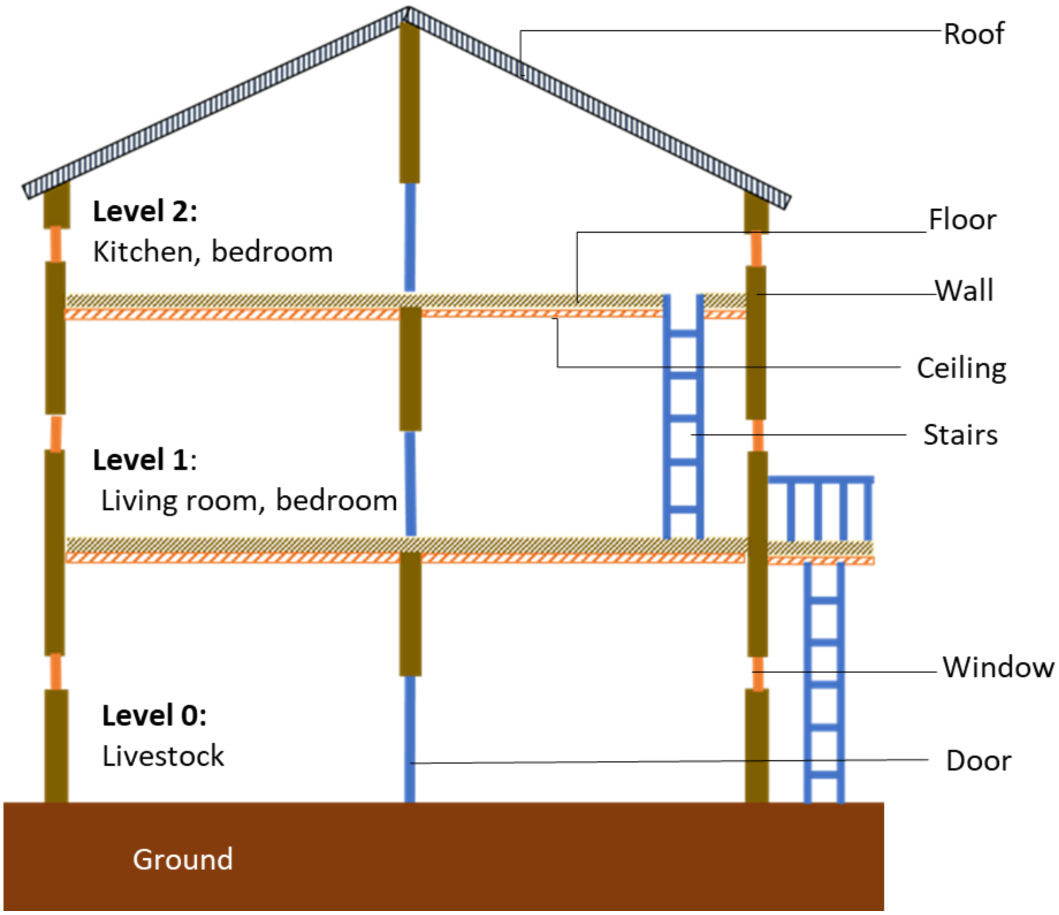

Supplement: S3 File — (PDF) [file pntd.0012036.s003.pdf]
