# Supplementary material for: Socio-ecological risk factors associated with human flea infestations of rural household in plague-endemic areas of Madagascar
Source: PLoS Negl Trop Dis. 2024 Mar 7;18(3):e0012036. doi: 10.1371/journal.pntd.0012036 (PMC10950221; doi:10.1371/journal.pntd.0012036)
Supplement: S4 File — (PDF) [file pntd.0012036.s004.pdf]

Additional file 4.

Table representing the output of the sensitivity analysis of the infestation at high risk for interhuman *Yersinia pestis* transmission using *m* upper and lower cut-off values.

Variables	Levels	Upper *m* cut-off value				Lower *m* cut-off value			
		aOR	p-value	Low CI	High CI	aOR	p-value	Low CI	High CI
Roof type	Elaborate (ref)								
	Primitive	3.05	0.26	0.48	25.7	3.32	0.13	0.74	17.36
Floor type	Mat (ref)								
	Other	0.19	0.14	0.01	1.48	0.23	0.13	0.04	1.18
Head of the household finished primary school	Yes (ref)								
	No	0.41	0.27	0.08	1.89	0.55	0.4	0.13	2.83
Any household member sleeping on the floor	No (ref)								
	Yes	1.34	0.7	0.29	6.09	0.93	0.92	0.23	3.58
Any household member sleeping under ITN	Yes (ref)								
	No	0.62	0.59	0.1	3.33	0.63	0.55	0.13	2.83
Ever used insecticide	Yes (ref)								
	No	3.94	0.11	0.82	24.19	2.06	0.29	0.56	8.19
Reported rodent presence	No (ref)								
	yes	12.42	0.05	1.5	305.93	4.23	0.08	0.92	23.67
Chickens indoors at night	No (ref)								
	Yes	4.3	0.08	0.92	25.69	3.56	0.06	1	14.07
Cows indoors at night	No (ref)								
	Yes	1.86	0.51	0.29	13.46	0.74	0.72	0.13	3.69
Pigs indoors at night	No (ref)								
	Yes	1.72	0.62	0.21	16.46	3.79	0.13	0.72	23.4
Village	ALA (ref)								
	AMB	0.18	0.23	0.01	2.75	0.41	0.46	0.04	4.27
	NAN	0.56	0.61	0.05	5.41	1.37	0.76	0.18	11.2
	SOA	0.05	0.05	>0.001	0.79	0.28	0.25	0.02	2.45

From the simulation of the average number of *Pulex irritans* per person required to sustain *Y. pestis* transmission noted *m*, a value equal to 7.43 (7.31, 7.55 95% CI) was found. Households that presented a flea index above *m* were categorized as at risk of *Y. pestis* transmission (n= 33/ 107). sensitivity analysis was performed using a generalized linear model with the upper and lower value of *m* 95% confidence interval as dependent variable. From the sensitive analysis, the results are robust to the 95% CI of the *P. irritans* index per person cutoff for *Y. pestis* transmission risk, as such cutoff changes do not change the distribution of households into low vs high risk. aOR: adjusted odds ratio, CI: confidence interval, ref: reference
