# Supplementary material for: Socio-ecological risk factors associated with human flea infestations of rural household in plague-endemic areas of Madagascar
Source: PLoS Negl Trop Dis. 2024 Mar 7;18(3):e0012036. doi: 10.1371/journal.pntd.0012036 (PMC10950221; doi:10.1371/journal.pntd.0012036)
Supplement: Attachment — Submitted filename: Review report_PNTD-D-23-00972.pdf [file pntd.0012036.s005.pdf]

PLOS Neglected Tropical Diseases submission number: PNTD-D-23-00972
Title: “Socio-ecological risk factors associated with human flea infestations of rural household in plague-endemic areas of Madagascar”

Responses to the review comments

Answers are in blue and bold character.

Methods

Reviewer #1: Nice use of candle trap!

Is the R code available with the data ? I would strongly recommend that it is, either as part of suppl data or at any of the servers with doi or github.

R code and datasets are available at the following link: https://osf.io/6mqux/?view_only=70d21dff44ce42bc8fc9bc45d8a9bf8c

Reviewer #2: Major critique:

Stepwise regression should almost never be used. The selection of variables to include in a regression model based on their individual p-values is normally bad practice, even though it is a practice that has been proposed and is still used (perhaps especially in some biomedical journals). There is now quite a bit of literature about why the approach is normally inappropriate, why it leads to analytic and statistical problems, and the different types of problems that are inherent in this approach.

While this article is in the "Journal of Big Data", the logic is relevant for smaller datasets as well and the bibliography lists many of the papers that discuss problems with the approach.

https://nam11.safelinks.protection.outlook.com/?url=https%3A%2F%2Fjournalofbigdata.springeropen.com%2Farticles%2F10.1186%2Fs40537-018-0143-6&data=05%7C01%7Cstephen.mugel%40emory.edu%7C50200de48a3d49f7a61108dbaec21c69%7Ce004fb9cb0a4424fbcd0322606d5df38%7C0%7C0%7C638295920955432410%7CUnknown%7CTWFpbGZsb3d8eyJWIjoiMC4wLjAwMDAiLCJQIjoiV2luMzIiLCJBTiI6Ik1haWwiLCJXVCI6Mn0%3D%7C3000%7C%7C%7C&sdata=ogD1Pt0OhysK%2B6%2F4%2BW5Qn3ARJ69I2wN3m0rsOIJCfwA%3D&reserved=0

Here are a few other accessible articles that likewise include references to the relevant literature:

https://nam11.safelinks.protection.outlook.com/?url=https%3A%2F%2Ftowardsdatascience.com%2Fstopping-stepwise-why-stepwise-selection-is-bad-and-what-you-should-use-instead-90818b3f52df&data=05%7C01%7Cstephen.mugel%40emory.edu%7C50200de48a3d49f7a61108dbaec21c69%7Ce004fb9cb0a4424fbcd0322606d5df38%7C0%7C0%7C638295920955432410%7CUnknown%7CTWFpbGZsb3d8eyJWIjoiMC4wLjAwMDAiLCJQIjoiV2luMzIiLCJBTiI6Ik1haWwiLCJXVCI6Mn0%3D%7C3000%7C%7C%7C&sdata=fY2SfyG3H3M1wqlL6nxO1squMdsK68kOT2%2FzAQZ4rwQ%3D&reserved=0

https://nam11.safelinks.protection.outlook.com/?url=https%3A%2F%2Fstatmodeling.stat.columbia.edu%2F2014%2F06%2F02%2Fhate-stepwise-regression%2F&data=05%7C01%7Cstephen.mugel%40emory.edu%7C50200de48a3d49f7a61108

dbaec21c69%7Ce004fb9cb0a4424fbcd0322606d5df38%7C0%7C0%7C638295920955432410%7CUnknown%7CTWFpbGZsb3d8eyJWIjoiMC4wLjAwMDAiLCJQIjoiV2luMzIiLCJBTiI6Ik1haWwiLCJXVCI6Mn0%3D%7C3000%7C%7C%7C&sdata=SC3L0erukQld%2B8jzPufFsbQ1z6zgX3D1UYEoxjuX0QA%3D&reserved=0

Briefly, a better approach to regression would begin with scientific hypotheses about which variables are likely important for the process that you are trying to model. Presumably, many of the variables from the survey were included in the survey because the researchers thought they could be important.

One of the larger problems with stepwise regression then comes from its deviance from the scientific approach. If you have a variable that you think should be important for the process you're modeling, and it is not showing a statistical association - that finding is of scientific value. It is equally as important (maybe even moreso) than a statistically significant finding. A related issue is that sometimes variables in a regression modify the association between other variables and the outcome of interest - EVEN WHEN they are not statistically significant. Conversely, another issue is that some variables that are not statistically significant on their own can become statistically significant in a regression model, after other factors/variables have been accounted for.

I suggest starting with the variables that you think should be important (hopefully based on scientific hypotheses or logic). Generate a table with their odds ratios and confidence intervals (or other relevant statistics - such as abundance/counts/risk ratios, etc.) Then include the model adjusted odds ratios and confidence intervals in the table as well. IF you have variables that are redundent, then it could be good to exclude all but one from the model based on that redundency (not based on p-values). You can often tell which ones are redundent by looking at how they co-vary (are they essentially always the same in the same households?), through logical reasoning, or by model behavior.

We changed our analytic approach in accordance with this advice. Our model now includes the parameters we hypothesized to be associated with flea abundance, and included only if they were not conceptually redundant, collinear, or too sparse across the measured villages. Indeed, it is valuable to demonstrate that hypothesized associations were not actually associated with the outcome, and we have expanded the discussion of these variables as well.

Another issue that I see has to do with the model that was used (binarly generalized linear model, which most folks would call a logistic regression). The approach begins with Equation 1 - which is an equation for the number of fleas per person required to sustain transmission, under a host of assumptions (including a major one of R0 = 1). I believe the authors need to either re-think this step or provide further justification for its use. It depends heavily on quite a lot of assumptions, and furthermore i don't think all of the assumptions or this approach are necessary. We already know that there is ongoing disease risk in this setting. Why rely on a theoretical model that would normally only be applied to larger populations? For example, R0 is normally only useful as a concept for larger, homogeneously mixing populations. From this equation, a continuous variable is generated. This continuous variable is then categorized as binary (0 or 1) based on a hypothesized threshold over which there is greater risk of infection. I see another problem with this step, in that risk is not binary and the threshold for high versus low risk appears relatively arbitrary.

My proposal then would be to model the abundance of fleas. This could also be done using a generalized additive model, but would instead be a type of count model (Poisson, negative binomial, etc., depending on the data). It would be possible and important to account for household occupancy still. The regression could account for repeat observations within households across nights and seasons using a random intercept at the household level (i.e. each household has its own intercept). Seasonal variation could then be measured in abundance, while accounting for the other factors that might be of importance. I believe that this would be a better analytic approach, and that it would still achieve the goal of this research: to indentify factors that contribute to risk of infection by rodent/flea-borne disease - with an inherent assuption that more fleas per person is tantamount to more risk of flea-borne disease per person.

Another approach could be to model 'm' from Equation 1 as a Gaussian GLM, but the approach that I suggest above (Poisson or negative binomial, with counts of fleas per house and a random intercept per house) would likely be more straightforward and would rely on many fewer assumptions.

You raise several excellent points regarding our analytic approach which we have integrated into our revision, improving the manuscript and message. Most importantly we have included a model that also assesses how risk factors are associated with flea abundance using logistic regression and a negative binomial distribution as you suggest and as the count data support. We have included both the flea abundance and the model relevant to epidemiologic data, and have discussed both, because these offer slightly different interpretations and meanings, both important. We argue that the threshold for high vs low risk is not arbitrary, but rather is rooted in modeling such a threshold for the average density of fleas to sustain transmission, which has been done previously (. https://doi.org/10.1073/pnas.0606831103 ; or https://doi.org/10.1086/429931). By invoking this plague context and the flea abundance models together, we believe this manuscript makes a more compelling case regarding important risk factors for P irritans infestation and its potentially epidemiologic impact.

Minor issues:

Line 73: "...plague is the most infamous." I don't doubt this, but since this is a scientific manuscript it is probably better to only include statements that can be supported, or to soften the statement. One solution could be to include: "...plague is arguably the most infamous" Or otherwise the authors could include a reference that shows that plague is in fact the most infamous (surveys?)

Change has been made to the sentence.

Line 79: State what is mean by 'humans are most vulnerable' Does it mean they are more likely to come into contact with rodents or fleas? To be infected? To have severe disease?

Change has been made to the sentence.

Line 103: Household level analyses are valuable and there could be household level interventions - but the results from household level analyses could also lead to community level interventions, whereby community engagement approaches community members with information about household level correlates of fleas.

Change has been made to the sentence.

Line 119: Does the fact that interviews were done in the dry season influence the results? Might respondents have answered differently if they'd been interviewed in the wet season? Is this also why there were very few male respondents?

Arguably, the amount of time spent at home and perceiving rodent movement, flea nuisance and thus insecticide use may differ depending on the season. However, most of the parameters such as household cleaning habits, livestock housing, sleeping habits and household size included in this study would remain unchanged.

The small number of male respondents reflects the cultural division of task in Malagasy culture, where female (the mother) is responsible for housework and thus more likely to be at home during interview times.

Line 130: How were the surveys developed? Were they based on pre-existing surveys?

The survey general layout has been developed from previous studies conducted by the Gillespie's lab in Madagascar (non-published).

Line 138: How were households selected for inclusion in this study?

Every household within the village qualified for the study. Households were selected when the head of the household was present during the visit and agreed orally to participate in the study. Investigators started at the center of the village and moved outwards.

Line 249: The authors probably mean 'multivariable analysis" instead of 'multivariate analysis'. A multivariate regression would mean that there were different outcomes being modeled. I believe here there is a single outcome being modeled, with muliple predictor variables in the model. This would be a multivariable regression.

Line 255: I'm not sure what is meant by "our analysis did not demonstrate statistical significance." It looks as though several variables are statistically significant.

Along the same lines, it would be better to present model-adjusted odds ratios (or rate ratios with a Poisson or negative binomial regression) and their confidence intervals. No p-values are needed if we have the effect size and confidence intervals, and these statistics are much more informative than the p-values. It is good to not rely too heavily on p-values as few people really understand them.

We considered the suggestions for our data analysis and the interpretation of the results

Results

Reviewer #1: Was the night irritation/bits truly linked to fleas. Were bedbugs considered? Often bedbug are not known and blame goes onto fleas. Not self-reporting bedbugs does not mean they not there.

The number of fleas collected in the study site as well as the answer obtained from residents may attest that most of the perceived nuisance is coming from flea bites. Though it is totally true that non-reporting bed bugs does not mean their total absence, we often observe in other study areas that bedbugs are a community problem, meaning that most of the residents would declare bedbugs if they were present.

I did not find the information about the animals within household, descriptive data.

The proportion and number of households keeping farm animals indoors at night are presented in table 1.

The floor mats and dirt makes perfect sense with pigs indoors. This is perfect for Pulex emergance. The lesser # of Ctenocephalides is likely that dogs would not be allowed inhouse unlike pigs that would be spending more time there, so depositing flea eggs on he floor mats to develop.

You OR for pigs indoors includes 1 and that is really unexpected for me, could you granulate the "livestock" what other livestock is there?

The proportion and number of households keeping farm animals indoors at night are presented in table 1.

What about daytime?

Poultry usually roam freely outdoors during the day and enter the home at dusk. Rabits and guinea pigs stay indoor all the time. Cows are usually held in outdoor enclosure or pasturing during the day.

Where are the pigs traditionally? Inside or outside during the day? Or are they allowed inside during the day?

Pigs are traditionally kept within enclosures during the day and night. The pigpen is most of the time installed indoor. Practice may vary between different region (https://doi.org/10.1016/j.prevetmed.2009.08.010)

Reviewer #2: Please see my comments in the methods section, which will have relevance to this section.

Conclusions

Reviewer #1: The recommendations are sound and completely fair given the data authors had. Authors should point that the fleas that emerge are only those that developed in the household, fleas don't jump off the animal . So what you detected is what developed within the trap. Placing the trap in areas where animals spent most of their time is beneficial, because they will usually not emerge (triggered by the light/heat) further then say 2 feet away.

We collected fleas that were blood fed (not discussed in the paper) which means that *P. irritans* would jump off their host. Furthermore, we only sampled in the bedroom because fire hazard would be a problem if a candle trap were placed in a room where animals are kept. A different type of trapping device may be more practical.

Pulex will be primary from pigs in this context, hence my question before about the pigs inside and time they spent there day/night. While you cannot go back you can provide cultural context beyond just saying they keep them in at night due to theft . I would be expecting that pigs might be simply allowed to be inside the house all the time and in fact rest there as well.

Study examining pig husbandry in various regions of Madagascar may give some indication regarding the time spent by pigs indoors. It showed that in a district of the central highlands, pigs "were either kept in the basement of their owner's house (53.1%), or in small pens with mud walls and thatched roof (42.0%)" and "61.7 % of pig owners reported allowing their animals to roam for food". Therefore, pigs may have an indoor -outdoor life, and may spend their daytime outside, but would spend their night within the house independently of fear of theft. (https://doi.org/10.1016/j.prevetmed.2009.08.010)

Reviewer #2: Please see my comments in the methods section, which will have relevance to this section

Editorial and Data Presentation Modifications

Reviewer #1: Make the data and code available via doi

Data and code are available at the following link and as additional file.

https://osf.io/6mqux/?view_only=70d21dff44ce42bc8fc9bc45d8a9bf8c

Reviewer #2: Please see my comments in the methods section, which will have relevance to this section

Reviewer #3: Review of the manuscript entitled "Socio-ecological risk factors associated with rural household human flea infestations in plague-endemic areas of Madagascar."

The manuscript is presenting a study with two main components. First, analysis of data from field data collections in Madagascar of human fleas in rural households across dry and wet seasons in 4 villages is done. This elucidates the association of possible risk factors with high flea infestation. Second, the researchers propose a model to estimate the minimum number of fleas per person necessary to sustain plague transmission once the pathogen enters the human-flea ecosystem.

Overall comment: this research adds important insights to the field and adds valuable information to the mounting evidence that the human flea could also be involved in plague outbreaks even if only in a minor role. The description of the environment Malagasy live in, in rural areas, and how they are affected by and deal with human flea infestations is very informative. I would suggest elaborating more on this to enable readers to understand the conditions better. Explaining the living conditions and what kind of livestock is usually kept where is important (such as broody hens in the bedroom).

Major comments:

In the background section:

Traditional housing is associated with flea infestation, so maybe add a short paragraph here on living conditions and traditional housing in rural Mada. % of people living with reed roofs, mats,

livestock below (which livestock species are in the houses), fleas are abundant, sometimes rooms/floors are abandoned due to flea infestation.

For Region Amoron'i Mania and Haute Matsiatra (regions where the study sites are located), 62.5% and 61.2 % of the houses have thatched roof, and 42.6% and 47.5% of the households use plant fiber mat cover on the floor, respectively. This information has been added to the discussion.

Table 4 belongs into the results section.

Table 4 has been moved to the results section.

Please discuss in more detail the use of the mats on the ground, namely as an affordable substitute for furniture (i.e. people sitting on the mats during meals, sleeping on mats, children playing on the mats). Following this it is important to also later discuss the difficulties of discouraging their use and suggest ways of improving the situation (exposing them to the sun, dusting with strong insecticide etc.). Also the possibility of government interventions such as IRS.

Woven plant mats have a strong cultural value for Malagasy people, and it is used for traditional rites as well as furniture. Regardless of the household floor type, it is common to sleep, receive guests and eat meals on mats that are not the one used to cover the floor. These mats are rolled and stored after use. In this study, the use of plant fiber mat as permanent flooring, covering the bare soil was associated to higher *P. irritans* infestation. We therefore believe that any program that would help the household to afford a different floor type would be readily welcomed by the population.

Previous study showed that insecticide treatment alone may not be efficient to reduce *P. irritans* burden in a village (https://doi.org/10.1371/journal.pntd.0007604) House improvement program would likely have more impact since the change of floor type would not allow infestation to take place although reinfestation may occur through time. Since house modification as a control strategy is expensive, our study allowed to identify the profile of households to be prioritized. Increasing the number of households with more durable and easier to clean floor type would likely benefit the entire community by reducing the flea infestation prevalence at a village level. As reported for programs targeting other vectors, house improvement generated other health benefits and increase the life quality of the inhabitants .

Please add to your limitations that trapping in the same type of room (i.e. bedroom) across all households would standardize data more.

Also, that trapping across 2 wet seasons but only 1 dry season may have affected the results on seasonality.

We only trapped twice for each site : once in dry season and once during the wet season.

Apart from lab studies to learn more about P. irritants, I suggest you also recommend research on possible interventions.

Possible intervention has been added to the discussion.

Minor comments:

Please consider changing the title to:

Socio-ecological risk factors associated with human flea infestations of rural household in plague-endemic areas of Madagascar.

Title changed accordingly.

Page 2 line 32 change to "risk factors associated with such…"

The change has been made.

Background:

Please add a sentence explaining the variable host specificity of fleas. (ref 10).

Host specificity explanation has been added.

Page 5 line 90-92: what do want to state by saying other plague reporting countries do not report this magnitude of human fleas? Do you mean that is why Mada may have more and more prolonged outbreaks? Explain please.

We only wanted to highlight the fact that the flea infestation is exceptional when compared to other countries. This observation alone does not allow us to draw any conclusions related to plague prevalence since other factors should be considered.

Page 6 line 126 informed oral consent or written consent?

Oral consent. The sentence has been modified.

Page 8 line 164: explain threshold septicemia.

Threshold septicemia is the minimal bacteria load per unit of blood volume that allows flea to get infected from a single blood meal.

Page 8 line 168 you need to define household size to avoid misunderstanding. People per household versus floor area in m2

Household size is the number of people living permanently in each household.

page 8 line 174: categorical variable levels like what? Can you give an example?

Please see in Table 1, for example for the variable "Respondent gender", the proportion of male respondent is less than 20%.

Table 1: did you ask how often insecticide was used?

We did ask a question about the frequency of insecticide use. The respondents answered that they use it as often as they need, without a clear frequency.

How did you deal with the connection between owning livestock and keeping livestock?

In table 1, for the variable "Livestock kept indoors at night", the "No" category(n=22/104) is the sum of households that don't own livestock at all (n=15) and the ones that own livestock but keep them in separate building at night (n=7).

Results:

Please explain clearer why keeping livestock would increase P. irritans density.

An explanation was added.

Page 10 line 192: women or men more likely to finish primary school?

According to World Bank, women are more likely to finish primary school in Madagascar.

Line 193 sleeping on the floor on what?

Sleeping directly on the floor without anything, or sleeping on a mat, blanket, rags, or mattress.

page 11 203 covered with a woven plant....

"A" added

Line 208 consider calling it corrugated iron sheet instead of metal sheet.

Metal sheet roof was replaced by corrugated iron sheet.

Line 209 ...mainly reported to be the house mouse....

Modification has been made.

Line 215 please stick to wet and dry or explain earlier that warm and wet and cool and dry goes together. Otherwise the reader cannot follow here.

Wet season has been added between parentheses. The answer we obtained referred more to temperature being warmer than to wet season.

Page 12: any info on what active ingredient is in the commonly sold insecticides?

Pyrethroid and Organophosphate when referring to brand names recalled by villagers.

Page 14 line 256 ..was found to be lower...

Modification has been made.

Line 259 ...found for households

Discussion

Why is table 4 in the discussion? Should be in the results section

Table 4 has been moved to the results section.

Page 17 line 307 delete Conversely

Modification has been made.

Consider mentioning a possible connection between the use of mats and thatched roofs, because they are both indicators of low socio-economic status.

A sentence has been added to the discussion about the use of locally sourced and more affordable housing materials.

Consider rewriting the livestock part in the discussion to clarify what species are usually kept per household (i.e. every hh has chickens), where they are kept etc.

This has been clarified in both the table and the text.

Page 20 line 363 delete susceptible

Line 379 present tense: demonstrate

Conclusion

Page 21 line 389: ...competence are needed... Line 391 change to: ...index. According to the model estimates this puts them....

This section has been rewritten.
