# Supplementary material for: Socio-ecological risk factors associated with human flea infestations of rural household in plague-endemic areas of Madagascar
Source: PLoS Negl Trop Dis. 2024 Mar 7;18(3):e0012036. doi: 10.1371/journal.pntd.0012036 (PMC10950221; doi:10.1371/journal.pntd.0012036)
Supplement: Attachment — Submitted filename: Response to reviewers comments.docx [file pntd.0012036.s006.docx]

Response to reviewer’s comments

We highly appreciate the reviewer’s comments and their contribution to improve the manuscript quality.

Please find below in blue font the detailed list of our responses to all review comments, and a description of the changes made in the manuscript.

Reviewer #2

Major:

A few of the variables (especially with regard to house materials) were not statistically significant in the models. In particular, this means that their confidence intervals crossed the 1.0 threshold, and that it isn't really possible to say whether those associations were negative or positive. This doesn't exclude the possibility of a true ecological association, and it is possible that the reason for not being statistically significant is because of a lack of power. There are other possibilities as well.

Line 365 - please soften this statement since we can't be sure whether the association was negative or positive, given the confidence intervals. There is a lot of uncertainty in that point estimate. You could mention that the odds ratio is large and positive, but that the confidence intervals crossed the threshold so that no certain statements can be made about the association between this variable and the outcome.

The sentence has been changed as follow:

Although the results did not achieve statistical significance and lack precision, the substantial effect size observed for primitive roof types (e.g., thatch) being linked to an increased likelihood of falling into the higher *Y. pestis* transmission risk category implies a potentially noteworthy role for this construction material. However, it is essential to note the need for further investigation to validate these preliminary findings.

Line 383: Do note that there was an effect of having chickens indoors and the abundance of fleas...

Indeed. Thank you for bringing up this finding that deserves more discussion. The following paragraph has been added to the discussion regarding livestock:

Although the odds ratios of keeping pigs and chicken indoors at night were non-significant with plague risk analysis (Model 2), the analysis of factors affecting flea abundance (Model 1) showed that keeping chickens indoors at night increases the odds of having *P. irritans* infestation (Table 4). Raising chicken was among the risk factor for house flea infestation in a study conducted in China (24) and chicken DNA was among the host genetic material detected in wild-caught *P. irritans* in DR Congo (47). In this study, 66.35% of the households raised chicken and more than 60% kept them indoors at night. Therefore, keeping those animals in separate structures may alleviate flea burden.

Line 398: We can't be sure from this analysis that a larger sample size would actually reveal the hypothesize association. If the authors wanted to, they could pursue a post-hoc power analysis and discuss those results along these lines. I would suggest just softening these statements about ORs that had incredibly broad CIs.

Also, I note from Table 4 that there is a positive association between reported insecticide use and the odds of having an 'infestation' of fleas in the house. I've seen similar results previously for other disease systems, where individuals who are experiencing lots of exposure to arthropods are most likely to use insecticides to address that exposure. The results from a cross sectional survey can then make the association appear causal in the opposite direction - where houses with more insects are the most likely to use insecticide (though the insecticide was unlikely to be the cause of the insect infestation). Regardless, I suggest rewording the statements from line 398 with this in mind, and also with the results from Table 4 in mind.

The sentence has been altered to reflect the reviewer’s suggestion to consider the large Cis:

Domestic insecticide use was highlighted as a common practice for flea control in our study. The model suggested that insecticide use against any household pest might be an important factor that influenced flea infestation. The elevated prevalence of *P. irritans* infestation may elucidate the necessity for employing domestic insecticides to mitigate the perceived nuisance as reflected in Table 2. Nonetheless, our analysis revealed an elevated adjusted odds ratio with wide confidence interval (Table 4), emphasizing the need for caution in interpreting the possible effect due to the considerable uncertainty. Although our model showed that chemical insecticides may have had a protective function against high flea abundance, there are concerns for the long-term efficacy of this method due to insecticide resistance.

Minor:

Line 110 - there is a mention that in Madagascar, during plague outbreaks, there are human flea infestations 'at magnitudes rarely found in other...' - I suggest specifying what is meant here explicitly. Perhaps something along the lines of:"...frequently infested by large numbers of human fleas..." This might help this statement be a bit more clear.

There was an error on that sentence, and it was changed as follow: “Furthermore, households in plague-endemic areas of Madagascar were frequently infested by a large number of human fleas at magnitudes rarely found in other countries reporting human plague outbreaks.”

Line 208: Please use "generalized" for the generalized linear model.

We modified general by generalized. Thank you.

Line 211 - there may be a typo in between "m. Seasonality"

Thank you, the typo has been addressed.

Lastly, I found the positive association between numbers of humans in a house and numbers of fleas in a house to be interesting, especially given that the population dynamics of these anthropophilic fleas should be associated with the numbers of hosts to which they have access. Perhaps this association is obvious, but I would at least mention it in the discussion because it is a valuable finding and could potentially push the referenced modeling work further (i.e. there should/may be feedbacks between human and flea population dynamics).

We appreciate that reviewer #2 made this remark. A new section was added to the discussion that reflects the potential implication of this positive association between household size and flea infestation.

Rodent presence was examined in this study, since rodents are the host of many flea species, including *P. irritans* (53). Interestingly, *P. irritans* has rarely been found infesting rodents in Madagascar and thus, might not be the primary host for this flea species (21,51). Analysis of homeowner responses concerning perceptions of flea nuisance (Table 2) suggests a potential scenario where this species is feeding on humans. Interestingly, we found a positive association between household size and *P. irritans* abundance. Households with more than four members have, on average, 1.92 times higher odds of experiencing P. irritans infestation (Table 4). Consequently, the larger family size may offer an increased opportunity for fleas to access blood meals and sustain a larger flea population. Moreover, in larger households, diverse activities may increase flea exposure. Similar trends were observed in Ethiopia regarding tungiasis (42,43), and in Bogota regarding flea-induced skin disorder (41), indicating higher risks for children going to school, from larger families, and those using public transportation. Research in China suggests that floor fleas can transfer between houses, especially in larger villages (24). Sampling fleas in shared spaces like schools, churches, and public transportation could be valuable for future investigations.
